# Emerging Therapeutic Strategies for Parkinson’s Disease and Future Prospects: A 2021 Update

**DOI:** 10.3390/biomedicines10020371

**Published:** 2022-02-03

**Authors:** Noha A. Gouda, Ahmed Elkamhawy, Jungsook Cho

**Affiliations:** 1 College of Pharmacy and Integrated Research Institute for Drug Development, Dongguk University-Seoul, Goyang 10326, Korea; noha.gooda@dongguk.edu (N.A.G.); a.elkamhawy@dongguk.edu (A.E.); 2 Department of Pharmaceutical Organic Chemistry, Faculty of Pharmacy, Mansoura University, Mansoura 35516, Egypt

**Keywords:** Parkinson’s disease, neuroprotection, neurodegeneration, novel target, stem cells, neurodegenerative biomarker, α-synuclein, mitochondrial dysfunction, oxidative stress, immunomodulation

## Abstract

Parkinson’s disease (PD) is a neurodegenerative disorder pathologically distinguished by degeneration of dopaminergic neurons in the substantia nigra pars compacta. Muscle rigidity, tremor, and bradykinesia are all clinical motor hallmarks of PD. Several pathways have been implicated in PD etiology, including mitochondrial dysfunction, impaired protein clearance, and neuroinflammation, but how these factors interact remains incompletely understood. Although many breakthroughs in PD therapy have been accomplished, there is currently no cure for PD, only trials to alleviate the related motor symptoms. To reduce or stop the clinical progression and mobility impairment, a disease-modifying approach that can directly target the etiology rather than offering symptomatic alleviation remains a major unmet clinical need in the management of PD. In this review, we briefly introduce current treatments and pathophysiology of PD. In addition, we address the novel innovative therapeutic targets for PD therapy, including α-synuclein, autophagy, neurodegeneration, neuroinflammation, and others. Several immunomodulatory approaches and stem cell research currently in clinical trials with PD patients are also discussed. Moreover, preclinical studies and clinical trials evaluating the efficacy of novel and repurposed therapeutic agents and their pragmatic applications with encouraging outcomes are summarized. Finally, molecular biomarkers under active investigation are presented as potentially valuable tools for early PD diagnosis.

## 1. Introduction

Parkinson’s disease (PD) is the world’s second most predominant progressive neurodegenerative disorder. PD is characterized by resting tremor, bradykinesia, muscle rigidity, coordination problems or temporary paralysis, dysarthria, and dysphagia. Other symptoms include gastrointestinal tract and bladder malfunction, sleep disturbances, and psychiatric manifestations. In addition to the loss of substantia nigra (SN) dopaminergic (DA) neurons, other neural pathways are also impacted by PD. The etiology of PD may be linked to gender, growing age, hereditary background, environmental factors, nutritional insufficiency, or brain damage [1]. PD is affecting more than 1% of the population above the age of 65 and predicted to be doubled by 2030 [2]. This disease now affects 10 million people worldwide, making it the 14th largest cause of death in the United States, with approximately USD 25 billion spent on PD treatment each year [3].

Numerous proteins and molecular pathways are implicated in the etiology of PD. The aggregation and deposition of misfolded or abnormal proteins, such as alpha-synuclein (α-Syn), either contribute to or act as a sign of dying DA neurons [4]. Some instances of PD have been linked to mutations in genes, such as leucine-rich repeat kinase 2 (*LRRK2*), ubiquitin C-terminal hydrolase L1 (*UCHL1*), Daisuke-Junko-1 (*DJ-1*), glucocerebrosidase (*GBA1*), and phosphatase and tensin homolog (PTEN)-induced kinase 1 (*PINK1*) [5,6]. In this review, current treatments and pathophysiology of PD are briefly introduced. In addition, novel and innovative approaches to develop disease-modifying interventions for PD treatment, targeting α-Syn, autophagy, neurodegeneration, and neuroinflammation, are addressed. Several immunomodulatory approaches and stem cell research currently in clinical trials with PD patients are also discussed. Finally, molecular biomarkers under active investigation are discussed as potentially valuable clinical tools for early PD diagnosis.

## 2. Current Treatments

There is currently no cure for PD. There are only pharmacological interventions to alleviate the related motor symptoms via reinstating striatal dopamine tone using dopamimetic drugs, such as dopamine precursor and DA agonists, and inhibitors of monoamine oxidase-B (MAO-B), catechol-*O*-methyl-transferase (COMT), and decarboxylase enzymes. Dopamine is synthesized via the conversion of L-tyrosine into L-DOPA through tyrosine hydroxylase (TH) and consequent decarboxylation of L-DOPA by DOPA decarboxylase. Both MAO-A and MAO-B (localized mainly in glial cells in the brain) metabolize DA [7,8]. Because dopamine cannot cross the blood-brain barrier (BBB), L-DOPA must be used in dopamine replacement therapy. The conversion of L-DOPA to dopamine occurs in the DA neurons located in the SN, ventral tegmental area, and hypothalamus of the brain [9].

Although these medications enhancing DA function in the SN do not halt the progression of PD, they can contribute to significant improvements in motor symptoms, particularly in the early stages of the disease [1]. Nevertheless, their effectiveness decreases with time and becomes less reliable in treating symptoms. L-DOPA is also transported to the other parts of the brain, leading to hallucinations and cognitive impairments. Moreover, long-term treatment may lead to considerable motor abnormalities and spontaneous movements, a condition known as L-DOPA-induced dyskinesias (LID). Combined administration of L-DOPA with a COMT inhibitor reduces peripheral L-DOPA turnover, increasing its plasma half-life and delivering L-DOPA to the brain in a more continuous manner. Alternatively, MAO-B inhibitors may be a better treatment choice for early PD, because of their favorable safety profile and potential neuroprotective properties. Many attempts are currently being undertaken to synthesize novel MAO-B inhibitors with neuroprotective properties, providing promising examples of multitarget-directed pharmacological interventions for PD treatment [10,11]. Low doses of glutamate antagonists have been shown to slow the progression of PD by reducing DA denervation in the striatum [12]. Anticholinergics which restore the balance between dopamine and acetylcholine in the striatum may be beneficial to reduce tremors associated with PD, especially in the early stages [13].

Surgery may be recommended for patients who demonstrate LID. Deep brain stimulation (DBS) is a nondestructive treatment often used for patients with L-DOPA-induced adverse effects, although it may be associated with substantial negative neuropsychiatric problems [14]. Another challenge with DBS is that the grafted hardware may disintegrate, migrate, or be damaged. A new approach known as magnetic resonance-guided focused ultrasound ablation has recently been introduced in the treatment of tremor-predominant PD, providing noninvasive and more accurate targeting of lesions in the thalamus [15]. Furthermore, non-motor symptoms of PD such as depression or anxiety, memory and cognitive impairments, and sleep problems can be treated with a wide range of drugs, including antidepressants, cholinesterase inhibitors, and sedative agents, depending on the symptoms. However, the clinical outcomes are not satisfactory in many patients [16]. Current approaches used in PD management are summarized in Table 1.

## 3. Pathophysiology

### 3.1. Genetic Basis

To date, more than 20 disease-causing genes and 90 separate risk-associated variations have been linked to PD [19]. Approximately 5–10% of patients have forms of the disease caused by mutations in these genes. At least 11 autosomal dominant genes, including *SNCA*, *PARK3*, *UCHL1*, *LRRK2*, and *VPS35*, and nine autosomal recessive genes, including *PRKN*, *PINK1*, *PARK7*, and *DJ-1*, have been identified [20]. Among the genes known to be associated with PD pathology, the most common and sophisticatedly linked genes are *SNCA*, *LRRK2*, *PRKN*, *PINK1*, and *GBA1*. It has been reported that the overexpression of α-Syn protein, which is encoded by the *SNCA* gene in humans, causes DA neuronal degeneration [21]. The dopamine storage was also found to be impaired by different mutations in the same gene, including A53T, A30P, and E46K. These mutations are the main reason behind the aggregation of Lewy bodies (LBs) causing DA neuronal death in the substantia nigra pars compacta (SNpc) [22]. Oligomers of α-Syn proteins can further activate the toll-like receptor 2 (TLR2) signaling pathway and eventually trigger microglial activation, resulting in neuroinflammation [23]. Several inflammatory mediators such as complement cascade proteins, cytokines including tumor necrosis factor-alpha (TNF-α), interleukin (IL)-1β and IL-6, chemokines, reactive oxygen species (ROS), and reactive nitrogen species are also released in this process. Normally, neurotrophic factors hinder α-Syn protein misfolding. However, due to the dependent depletion of these growth factors in PD, accumulation and aggregation of α-Syn proteins increase inside the nerve cells [24].

*LRRK2*, a gene encoding leucine-rich repeat kinase 2, is the most commonly known cause of familial and sporadic PD [21]. Mutations in genes including *SNCA* and *LRRK2* are reported to be risk factors for sporadic PD. In fact, α-Syn LB pathology, the typical characteristic of sporadic PD, is observed predominantly in *SNCA* and *LRRK2* carriers [25]. Moreover, accumulating evidence supports a role for *LRRK2* in lysosomal biology, modulating membrane and vesicle trafficking and autophagy [26].

The *PRKN* gene encodes parkin, an E3 ubiquitin ligase, which plays a critical role in ubiquitination. This protein mediates the clearance of damaged mitochondria mainly via autophagy. Thus, it is widely believed that *PRKN* mutations are associated with mitochondrial dysfunction in PD [27,28,29]. Similarly, PTEN-induced kinase 1 (PINK1), a mitochondrial serine/threonine protein kinase encoded by *PINK1* gene, also controls elimination of dysfunctional mitochondria [30]. Mutations in *PINK1* and *PRKN* genes are associated with one form of autosomal recessive early-onset PD.

Mutations in the *GBA1* gene (L444P and N370S) are the most common genetic risk factor for sporadic, early-onset PD, particularly accompanied by rapid cognitive decline with LB [31]. These genetic abnormalities were also reported to cause increased protein aggregation and lysosomal malfunction [32]. The detailed pathophysiological roles of these PD genes in the regulation of mitochondrial function and the autophagy-lysosomal system are discussed in the following section.

### 3.2. Mitochondrial Dysfunction and Oxidative Stress

Mitochondrial dysfunction plays a key role in the pathogenesis of PD. The collective evidence from various experimental models and PD patients highlighted the disruptions of mitochondrial dynamics and bioenergetic impairments in PD, resulting in increased levels of ROS and intracellular calcium concentrations, decreased ATP production, and excitotoxicity-mediated neuronal damage [28]. Dysregulation of the mitochondrial dynamics affects mitochondrial fission, fusion, transport, mitophagy, and biogenesis [28,33]. In addition, α-Syn oligomerization and aggregation within the mitochondria is one of the main causes for the mitochondrial fragmentation [34]. PINK1 is required for parkin recruitment for the ubiquitin-proteasome system (UPS) degradation pathway [35,36]. Mutations in the *PINK1* and *PRKN* genes were also reported as major players in deregulated mitophagy, while a mutation in the *DJ-1* gene, which encodes for cytosolic antioxidant proteins protecting neurons from oxidative damage, also leads to neurodegeneration in PD [37].

Mitochondria particularly regulate and balance Ca^2+^ influx and efflux through ligand-gated glutamate receptors such as N-methyl-D-aspartate receptors (NMDAR) and voltage-dependent ion channels [38]. Neurons rely nearly entirely on mitochondrial oxidative phosphorylation for ATP synthesis, and mitochondrial Ca^2+^ uptake ensures activity-dependent cellular energy metabolism control. Any modest changes in Ca^2+^ homeostasis might have negative implications, leading to alterations in physiological neuronal activity [39]. In particular, *PINK1* and *PRKN* are two PD-associated genes that affect mitochondrial Ca^2+^ influx pathways. *PINK1* controls Ca^2+^ efflux from mitochondria via the Na^+^/Ca^2+^ exchanger, whereas *PRKN* activates voltage-dependent ion channels, allowing Ca^2+^ diffusion across the outer mitochondrial membrane. Accordingly, *PINK1* deficiency causes mitochondrial Ca^2+^ overload and subsequent production of reactive oxygen species (ROS), ultimately making neurons prone to death [35].

Moreover, the *PINK1* and *PRKN* genes play significant roles in adaptive immunity by suppressing mitochondrial antigen presentation, suggesting that autoimmune mechanisms may contribute to the etiology of PD [27]. Intestinal infection with Gram-negative bacteria in *PINK1* knockout mice increases the release of pro-inflammatory cytokines, activates mitochondrial antigen presentation, and induces autoimmune mechanisms provoking the activation of cytotoxic mitochondria-specific CD8+ T cells. These findings support the role of *PINK1* as a modulator of the immune system [29].

### 3.3. Autophagy-Lysosome System Dysfunction

Non-functional and anomalous proteins are abolished by one of three processes: the autophagy-lysosomal pathway, the UPS, and molecular chaperones [40]. Autophagy plays a crucial role in the progression of PD because it is responsible for delivering misfolded proteins and impaired organelles to the lysosome for degradation via different pathways, including macro-autophagy, micro-autophagy, and chaperone-mediated autophagy. Impairments in these pathways result in deposition of protein aggregates, which is a common pathophysiological characteristic of PD [41]. Similar to mitochondria, UPS functioning is usually regulated by *PINK1*, *PRKN*, *DJ-1*, and *UCHL1* genes. Abnormally aggregated protein affects the chaperone-mediated autophagic system as well as UPS, which influences neuronal function and axonal transport [42].

Although the *LRRK2* literature on autophagy is vast, the direction in which *LRRK2* mutations influence the autophagic system has been referred to as unclear or contradictory. However, following a detailed analysis of *LRRK2* phenotypes linked to autophagic flux, it is clear that *LRRK2* mutations, notably the G2019S and R1441C, function differently and at various phases of the autophagy process. The G2019S mutation in *LRRK2* is consistently related with increased kinase activity and decreased autophagic flux, whereas the second most common mutation, R1441C, is associated with decreased autophagic flux, with lysosomal activity being especially affected [43]. In addition, the *ATP13A2* (*PARK9*) gene, which is altered in some forms of early-onset PD, has been proposed as an autophagy-lysosome pathway regulator. The *ATP13A2* deficiency is linked to lysosomal dysfunction, autophagy impairment, and buildup of or Syn A53T, all of which are likely factors of PD pathogenesis [44]. Furthermore, *GBA1* mutation causes widespread alterations in lysosomal architecture and function, as well as an increase in the accumulation and release of oligomeric α-Syn protein. These findings support that a number of PD-associated genes, including *LRRK2, ATP13A2*, and *GBA1*, are implicated in the regulation of lysosome function associated with PD pathology [45].

**Figure 1:**
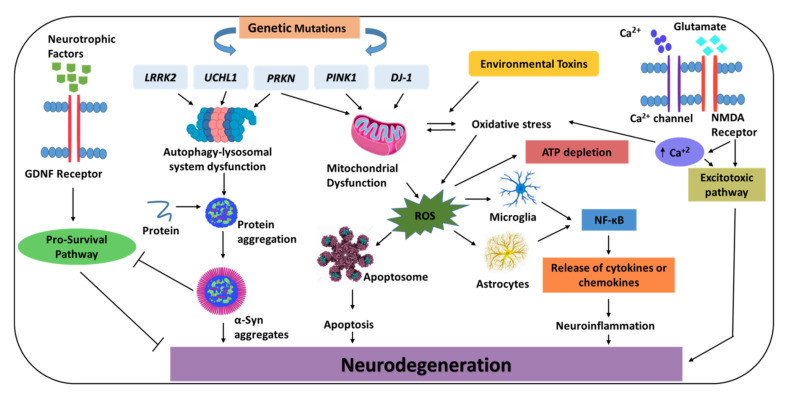
Molecular pathways involved in the pathophysiology of Parkinson’s disease (PD). Common pathogenic mechanisms in PD, including genetic mutation, defective protein clearance, mitochondrial dysfunction, loss of trophic factors, alterations of intracellular Ca^2+^ homeostasis, and neuroinflammation, are illustrated. Enhanced signaling pathways are indicated by solid arrows, and suppressed pathways are by blocked arrows. GDNF: glial cell-derived neurotrophic factor, *LRRK2*: leucine-rich repeat kinase 2, *UCHL1*: ubiquitin C-terminal hydrolase L1, *PRKN*: parkin, *PINK1*: phosphatase and tensin homolog-induced kinase 1, *DJ-1*: Daisuke-Junko-1, α-Syn: alpha-synuclein, ROS: reactive oxygen species, TNF-α: tumor necrosis factor-alpha, IL-1β: interleukin-1β, IL-6: interleukin-6, NMDA: N-methyl-D-aspartate.

As summarized in Figure 1, there are various hypothesized causes for neuronal death in PD, although not all have been thoroughly investigated. Protein aggregation in LBs, mutations in genes regulating autophagy and mitochondrial function, oxidative stress, and neuroinflammation are some of the key pathways underlying neuronal death in PD. The discovery of disease-modifying agents appears to be on the horizon as the understanding of the etiology of PD improves and novel therapeutic targets are being identified.

## 4. Novel Therapeutic Strategies in PD Management

### 4.1. Targeting α-Syn Aggregation

#### 4.1.1. α-Syn Misfolding Inhibitors

The aggregation process of α-Syn is highly complicated and reliant on environmental variables, so developing effective measures to inhibit this process has proven to be difficult. Furthermore, the toxic species of α-Syn has yet to be determined, which might restrict the therapeutic efficacy of these approaches [34]. However, some heat shock proteins (HSPs), particularly HSP70 and HSP104, were efficient in halting α-Syn aggregation in vitro and in vivo via partially stabilizing the transient folding intermediates, in an ATP-independent fashion, to sustain cellular proteostasis in stressful conditions [46]. In addition, natural α-Syn aggregation inhibitors, such as curcumin (a culinary spice), showed a substantial neuroprotective effect [47,48].

Small-molecule α-Syn inhibitors (SMSIs) are currently an intense area of research focus. NPT200-11 and NPT088 are two representative candidates currently undergoing clinical trials. Upon administration of NPT200-11 in a transgenic mouse model of PD, α-Syn aggregation in the cortex and neuroinflammation (astrogliosis) were reduced. NPT200-11 also enhanced the motor performance and normalized dopamine transporter (DAT) levels in the striatum. Furthermore, NPT200-11 is orally bioavailable and can penetrate the brain [49]. NPT200-11 completed a phase I clinical trial: it demonstrated a satisfactory safety profile and was well-tolerated at several doses (NCT02606682). NPT088 was suggested to bind to α-Syn, amyloid-beta, and tau aggregates (The latter two are implicated in Alzheimer’s disease (AD) and frontotemporal dementia), reducing the deposition of proteinase K-resistant proteins [50]. Furthermore, NPT088 reduced protein aggregation in a PD model and was tested in a phase I clinical trial in patients with mild-to-moderate AD. NPT088 was generally safe and well-tolerated (NCT03008161). However, it had no impact on brain plaques, tau aggregates, or AD symptoms [51]. The development of SMSIs for PD is highly needed in the next few years, considering the positive clinical findings of NPT200-11.

#### 4.1.2. Antisense Oligonucleotides (ASOs)

Another way to decrease *SCNA* (α-Syn gene) expression is to use ASOs to increase the breakdown of α-Syn messenger RNA levels (mRNA), delaying PD onset and/or changing the progression of the disease. Uehara et al. developed an amido-bridged nucleic acid (AmNA)-modified ASO targeting the α-Syn gene with enhanced stability and cellular uptake in vivo. AmNA-ASO effectively inhibited α-Syn at the mRNA and protein levels. Moreover, AmNA-ASO treatment improved the neurological abnormalities in a mouse model of PD expressing human wild-type α-Syn gene [52]. Another study discovered that intranasal delivery of small interfering RNA sequences selectively targeting serotonin (5-HT) or norepinephrine neurons and ASO conjugated with the triple monoamine reuptake inhibitor indatraline lowered the endogenous α-Syn mRNA and protein levels in the brainstem monoamine nuclei of mice [53]. In the rodent preformed fibril (PFF) model, ASO reduced the production of α-Syn, leading to the prevention of DA cell dysfunction. Moreover, the ASO showed broad activity and distribution throughout the non-human primate brain with a corresponding decrease in α-Syn levels in the cerebral spinal fluid (CSF) [54].

Intrabodies are another promising approach. Intrabodies are antibodies that have been designed for intracellular expression and can be directed to a specific target antigen present in various subcellular locations. They specifically bind with α-Syn monomers and block their oligomerization. For example, intracellularly expressed as intrabodies, anti-α-Syn nanobodies fused to a proteasome-targeting proline, aspartate or glutamate, serine, and threonine (PEST) motif can modify α-Syn monomeric concentrations with high affinity and specificity [55]. Another study showed that intrabodies might exert neuroprotective effects by disrupting the aggregation-prone region, the non-amyloid-β component [56]. Compared to immunotherapies, intrabodies require direct delivery to the central nervous system (CNS) through viral vectors [55]. Two proteasome-directed nanobodies selectively targeting α-Syn were delivered and shown to restore striatal dopamine tone and enhance motor function in the α-Syn-based PD model [55]. Another challenge facing their clinical application is achieving effective levels in the CNS for prolonged periods.

An alternative technique with a broad spectrum of activity targeting both prion protein and α-Syn oligomer involves structure-dependent epitopes. One example is the oligomer modulator anle138b. It has been demonstrated to have neuroprotective effects in vitro and in vivo, through structure-dependent binding to pathological aggregates of prion protein and α-Syn with promising oral bioavailability and BBB penetration [57]. A phase I clinical trial is currently recruiting patients with mild-to-moderate PD to demonstrate the safety, tolerability, pharmacokinetics, and pharmacodynamics of multiple oral doses of anle138b (NCT04685265).

#### 4.1.3. Beta2-Adrenoreceptor (β2AR) Agonists

The earliest clinical attempts to use β-adrenergic agonists in PD started in the early 1990s, when some open-label trials assessed the β2AR agonist salbutamol (a brain-penetrant asthma drug) [58,59]. Follow-up research in 2003 supported β2AR agonists to work in synergy with _L_-DOPA [60]. Unbiased screening of endogenous gene expression suggested a novel mechanism by which β2AR agonists act as regulators of the *α-Syn* gene, reducing its expression and consequent accumulation and deposition. Over the 11-year follow-up, salbutamol was associated with a reduced risk of developing PD. Conversely, propranolol (a β-adrenergic antagonist) correlated with increased risk [61].

These findings were further supported by evidence from Norwegian and Israeli populations suggesting a reduced risk of PD in patients treated with β2AR agonists and increased risk with β2AR antagonists [62]. Moreover, the data from the Israeli group showed that the beneficial effects of β2AR agonists depended on their dose-duration curve [62]. However, recent research in the American population found no link between β2AR medication and PD [63]. The immunomodulatory effect of β2AR agonists was suggested to be the mechanism of action behind the neuroprotection because the production of trophic factors or other proteins involved in glutamate-induced excitotoxicity is mediated by adrenergic stimulation of astrocytes [64]. Furthermore, stimulation of β2AR in the peripheral immune cells may control T cell activation by reducing the generation of pro-inflammatory cytokines. Moreover, norepinephrine promotes regulatory T cells (Tregs), which physiologically suppress T helper type 1 (Th1) cells, indicating that such a mechanism may be a promising therapeutic target in PD [47,65].

#### 4.1.4. Lymphocyte-Activation Gene 3 (LAG3) Receptor

LAG3, also known as CD223, is a transmembrane protein containing four extracellular immunoglobulin (Ig)-like domains (D1–D4) along with an intracytoplasmic portion [66]. As an immunological checkpoint receptor, it regulates T cell immune reaction and immune homeostasis by limiting T cell stimulation and proliferation [67]. Although the function of LAG3, which is highly expressed on neuronal cells and microglia, is mainly unknown, it has recently been implicated in PD pathophysiology, specifically in α-Syn transmission. Among three identified transmembrane protein candidates, LAG3 exhibited superior selectivity for binding to exogenous α-Syn PFFs in comparison with α-Syn monomers. The binding of α-Syn-biotin PFF to LAG3 led to α-Syn PFF endocytosis and neuronal transmission, spreading neurotoxicity [68].

LAG3 deletion decreased α-Syn neuronal transmission and α-Syn PPF-induced neurotoxicity in human A53T α-Syn transgenic neuronal cell cultures along with a reduction in intracellular Ca^2+^ levels, implying a role for LAG3 in human α-Syn pathology. In addition, LAG3 overexpression enhanced α-Syn phosphorylation, which is also implicated in PD pathogenesis [69]. In addition, given its role in immune control, LAG3 may play an essential role in the neuroinflammatory processes that contribute to PD pathogenesis. A recent study in the Chinese population found that serum LAG3 levels were considerably greater in PD patients than gender- and age-matched controls and patients with essential tremor [70], suggesting a potential role of LAG3 as a promising molecular biomarker of PD.

In summary, multiple lines of evidence support the critical importance of α-Syn clearance in PD management. We reviewed advances in current trials to alleviate pathological α-Syn species in PD, including blocking pathways implicated in α-syn aggregation at the transcription level (e.g., ASO) or hindering propagation mechanisms that underlie the cell-to-cell transmission of α-Syn between neighboring neurons (e.g., LAG3 deletion). The significance of potential disease-modifying therapeutics targeting pathological α-Syn species (e.g., SMSIs) was also discussed.

### 4.2. Enhancing Autophagy

#### 4.2.1. Mammalian Target of Rapamycin (mTOR) Signaling

The mTOR signaling pathway regulates several steps of the autophagy [71]. Both activation and deactivation of mTOR signaling are implicated in the various stages of PD. Autophagy, as previously stated, is essential for α-Syn degradation. α-Syn overexpression inhibits autophagy by increasing mTOR activity. Expression of the mTOR protein was reported to be enhanced in the temporal cortex of patients with α-Syn accumulation, whereas rapamycin (an mTOR inhibitor) reversed the enhanced mTOR activity produced by α-Syn overexpression [72]. RTP801/REDD1, on the contrary, is a stress-related protein that markedly inhibits mTOR activity, resulting in neuronal loss. RTP801 was found to be increased in SNpc neurons of PD patients [73]. Rapamycin inhibits RTP801 translation and, consequently, mitigates mTOR suppression, resulting in the preservation of Akt phosphorylation (an mTOR upstream kinase) for its pro-survival function [74,75]. Rapamycin was initially investigated in conjunction with L-DOPA in an animal model of PD. It auspiciously prevented increased mTOR activity and reduced dyskinesia caused by L-DOPA [76]. Therefore, in addition to autophagy induction to increase α-Syn degradation, inhibition of mTOR signaling by rapamycin provides a scientific rationale for L-DOPA therapy in PD. Rapamycin protected neurons in PD models by increasing autophagy flux and hindering RTP801 translation via mTOR inhibition [77].

Organic substances, such as curcumin [78] and piperine [79], also block mTOR and enhance autophagy, thereby rescuing neurons in the PD cellular model [71,80]. However, mTOR-dependent autophagy enhancers may impair cell growth because mTOR signaling controls cell proliferation and viability. Moreover, mTOR is required for cellular processes, such as synaptic plasticity and memory formation and retention [81]. To overcome the unfavorable effects of mTOR inactivation, introducing small molecules that increase autophagy activity without inhibiting mTOR may be beneficial for PD treatment. In this context, lithium [82,83], trehalose [84], corynoxine B [85], a synthesized curcumin derivative termed C1 [86], in addition to sodium valproate and carbamazepine (mood-stabilizing agents), were found to activate autophagy in vitro independent from mTOR with an increase in α-Syn degradation. An ongoing phase I clinical trial (NCT04273932) is investigating the effects of lithium therapy in PD [87].

Small molecules have recently been used to target PD by altering mTOR pathways. RTB101 is an inhibitor of the target of rapamycin complex 1 (TORC1). In preclinical models, the inhibition of TORC1 stimulated autophagy, protected against neurodegeneration, and improved motor function [88]. RTB101 treatment lowered glucosylceramide levels (the primary substrate of the lysosomal enzyme glucocerebrosidase (GCase)), indicating that RTB101 increases GCase activity, which can deaccelerate PD progression [14]. Interim data from three cohorts in a phase Ib/IIa study demonstrate that RTB101 is well-tolerated, crosses the BBB, and reaches the CSF in concentrations observed in preclinical models to inhibit the activity of TORC1 and induce autophagy in neuronal cells. RTB101 can be used alone or in combination with sirolimus (another mTOR inhibitor). Findings from the 4-week phase Ib/IIa trial of the RTB101 (300 mg) in combination with sirolimus (4 mg) once weekly were expected in 2020, but have been delayed due to the impact of coronavirus disease 2019 (COVID-19).

#### 4.2.2. Inhibition of Cellular Homolog of ABL1 (c-Abl)

The c-Abl belongs to the ABL (Abelson murine leukemia virus oncogene) family of tyrosine kinases, located in the cytoplasm and nucleus. It has diverse physiological roles, such as cell growth, cytoskeleton dynamics, DNA repair, receptor endocytosis, autophagy, and cell survival [89,90]. The biological importance of c-Abl in the CNS includes neurite outgrowth, synapse formation, and neurogenesis, in addition to cerebellar development. DA stress and DA neurotoxins activate c-Abl tyrosine kinase, leading to phosphorylation of E3 ubiquitin ligase PRKN, which subsequently causes the inhibition of PRKN ubiquitination and its protective function [91]. Activated c-Abl tyrosine kinase also downregulates peroxisome proliferator-activated receptor-gamma coactivator one alpha (PGC-1α), leading to mitochondrial dysfunction [92]. Furthermore, c-Abl promotes the proteolytic cleavage of protein kinase C (PKC) delta, increasing its activation, which drives the mitochondrial apoptotic pathway, resulting in cell death [93]. Alternatively, c-Abl inhibition deactivates microglial cells and decreases their release of pro-inflammatory mediators. Moreover, it enhances α-Syn clearance via increased autophagy [94], implying a role for c-Ab1 inhibition in PD management. Furthermore, c-Abl was activated in brain specimens from PD patients, indicating a pathophysiological involvement in PD [95].

To date, three c-Abl inhibitors (imatinib, nilotinib, and bafetinib) have been studied as possible disease-modifying PD treatments. However, due to imatinib’s insufficient BBB penetration and active transport out of the brain through the efflux transporters ABCG2 and ABCB1, it is likely that considerably high doses will be required to achieve the necessary results [96]. Nilotinib, a brain penetrating c-Abl inhibitor, has been approved by the Food and Drug Administration (FDA) to treat leukemia, but not PD [97]. Nevertheless, nilotinib raised the amounts of important autophagy proteins, such as light chain protein-3 chain I and II (LC3-I/II), PRKN, beclin-1, p62, and autophagy-like proteins in animal models of neurodegeneration, suggesting a role in α-Syn elimination via autophagy. Nilotinib significantly increased microRNAs (miRNAs) that control specific ubiquitination and autophagy genes in the CSF of PD patients in clinical trials [98]. In a phase II clinical trial in patients with moderately advanced PD (NCT03205488), nilotinib displayed an acceptable safety and tolerability profile [99]. However, because of the limited CSF exposure and poor effectiveness leaning to the negative direction, further exploration in PD was halted. Nilotinib concentrations in the CNS are limited owing in part to efflux transport via ABCG2 and ABCB1 [100]. Recent data suggest that bafetinib (INNO-406), a second-generation c-Abl inhibitor, achieves CNS concentrations almost thousands-fold higher than nilotinib and can reach CSF concentrations 10% higher than plasma concentrations [101].

The issue of adapting existing c-Abl inhibitors to PD is not limited to BBB penetration. Another issue is their specificity. Imatinib, nilotinib, and bafetinib are type-II kinase inhibitors [100,102]. Imatinib and nilotinib can block other tyrosine kinases, such as platelet-derived growth factor (PDGFR) or KIT [102]. Bafetinib also inhibits several kinases other than c-Abl, including diverse forms of Src, KIT/PDGFR, ephrin receptor families, Zak, and the discoidin domain receptors (DDR1 and DDR2) [103]. Although bafetinib was superior to imatinib or nilotinib in brain penetration, its off-target inhibition may have intolerable adverse effects. Despite these obstacles, there is an increasing demand and necessity for the future expansion of c-Abl inhibitors with improved BBB penetration and target-specificity to treat PD.

#### 4.2.3. RhoA-ROCK Signaling

RhoA is a signaling protein that plays a role in various physiological processes, including actin production, membrane trafficking, and inflammation. ROCK I and II are the major downstream effectors of RhoA [104]. RhoA/ROCK signaling may cause DA loss by activating microglial cells [104,105]. Activation of Rho kinase increases ROCK activity in microglia, which elevates ROS levels and, subsequently, the release of inflammatory cytokines [106]. RhoA-ROCK can cause DA loss via cofilin inhibition [107]. Cofilin is an actin-binding protein required for actin filament depolymerization and, consequently, inhibition of actin filament elongation. Rho abolishes cofilin’s actin-binding function, boosting actin filament polymerization. Growing findings imply a link between PD and actin dynamics [105] due to the discovery of actin in cytoplasmic α-Syn aggregates [108]. Accordingly, ubiquitous RhoA/ROCK is thought to play a role in PD. Brain samples from the frontal cortex of individuals with atypical PD syndrome (progressive supranuclear paralysis) demonstrated considerably higher increased levels of RhoA effectors [109]. Results from well-studied ROCK inhibitors, such as fasudil [110,111], Y-27632 [112], and statins [113] suggest that ROCK may be a promising therapeutic target for subsequent translational trials in neurodegenerative disorders. Fasudil repressed Rho kinase in the 6-hydroxydopamine (6-OHDA) mouse model of PD, resulting in a neuroprotective effect [111]. In paraquat-treated flies, Y-27632 stimulated the PRKN-mediated mitophagy pathway, resulting in neuroprotective consequences [112]. In the 1-methyl-4-phenyl-1,2,3,6-tetrahydropyridine (MPTP)-mouse model, simvastatin increased locomotor performance and protected DA neurons. Statins also inhibited the accumulation of α-Syn in vitro and in vivo [114,115]. Simvastatin is being tested as a neuroprotective PD treatment in a phase II trial (NCT02787590). The researchers anticipated that a 1-month low-dose phase of oral simvastatin, followed by a 23-month high-dose phase, could decrease the progression of PD. However, they found that simvastatin failed to slow the progression of PD [116]. Another placebo-controlled, double-blind, phase II study of lovastatin (NCT03242499) showed that a high dose of intensive lipid-lowering lovastatin was well-tolerated and associated with a tendency of decreased motor symptom deterioration in individuals with early-stage PD [117]. A larger, long-term follow-up study is needed in the future to validate this conclusion. Inhibiting RhoA/ROCK signaling is a viable strategy for neuroprotection in PD. However, more research is required to understand the multiple pathways of RhoA and its effector, ROCK.

#### 4.2.4. Lysosomal Acid Sphingomyelinase Enzyme (ASMase)

Sphingomyelin (SM) is the most prevalent cellular sphingolipid, with widespread distribution throughout mammalian tissues and high levels in the CNS. SM is a necessary component of the plasma membrane (PM), and critical for cell activity. SM metabolic enzymes activity maintains the equilibrium between SM synthesis and degradation and regulates the cellular SM content; sphingomyelin synthases (SMSs) and sphingomyelinases (SMases) maintain cellular homeostasis by modulating the levels of SM and ceramide (Cer) [118]. There are several forms of SMase, categorized into alkaline, neutral, and acidic SMase, which differ by pH optimum and subcellular location. ASMase is a well-characterized SMase with an optimal pH of 5 and predominantly located in the lysosomes, where its primary function is to degrade SM to generate Cer and phosphocholine [118,119].

A founder mutation (p.L304P) in the lysosomal gene sphingomyelin phosphodiesterase 1 (*SMPD1*), which encodes ASMase, can lead to abnormalities in the lysosome functions with disruption in α-Syn clearance and an increased risk for PD [120]. Moreover, dysregulated SM metabolism affects neurotransmission in PD [121]. Model membrane studies revealed α-Syn aggregation in SM-enriched regions with disruption of PM architecture. Changes in the structure of PM led to a disruption in membrane fusion, which affected the neurotransmitter exocytosis [122]. *SMPD1* mutations have been linked to an increased risk of PD or synucleinopathy in seven independent cohorts: two of Ashkenazi Jewish (AJ) origin [120,123], two Chinese [124,125], two European [126,127], and one North American [128]. Another study found that two AJ mutations, p.L302P and p.fsP330, are associated with PD. Both mutations lead to a lack of ASMase lysosomal distribution [129]. With the findings indicating that reduced ASMase levels result in α-Syn deposition, it is possible to speculate that all these abnormalities increase the risk of PD by inducing limited lysosomal localization of ASMase, concomitant with the accumulation of α-Syn via an unknown mechanism [129].

Another concept addressing the role of the SM metabolic pathway in PD etiology links the SM cycle to the inflammatory pathways. Translocation of nuclear factor-kappa B (NF-κB) to the nuclei of DA neurons in PD generates free radicals, which promotes SM hydrolysis, Cer generation, and apoptosis [130]. In the hippocampus of MPTP-induced PD rats, there was an increase in inducible nitric oxide synthase (iNOS) protein expression and a reduction in neutral SMase protein expression [131].

SMases and TLR4 have been correlated with neuroinflammation and PD. TLR4 deficiency increased neutral SMase protein expression and its enzymatic activity in the midbrain, with substantial delocalization of SMase from the cell membranes, in wild-type and TLR4-deficient mice treated with MPTP. This led to a reduction in SM species and a substantial rise in Cer levels. Additionally, treatment with MPTP in TLR4-deficient animals reduced unsaturated SM species and raised the saturated to unsaturated SM ratio. Saturated fatty acids harden SM and may lead to synaptic flexibility loss. In the midbrain tissues of TLR4 knockout mice, a reduction in both heavy neurofilaments and glial fibrillary acidic protein (GFAP) was noticed, and mice were more sensitive to MPTP injection. Accordingly, TLR4 was found to play a role in altering SM metabolism in MPTP neurotoxicity. A detailed understanding of the implication of TLR4 stabilization over SM metabolism would be desirable [132].

To sum up, pharmacological manipulations of autophagy may slow down neurodegeneration associated with PD. Nevertheless, as previously stated, the precise pathways driving autophagy defects may differ depending on the underlying etiology of the disease (e.g., mutations in one versus another specific gene causing PD). The therapeutic benefits of several small molecules acting as autophagy modulators targeting both mTOR-dependent and -independent pathways have been studied, and emerging clinical trials are showing some promising results. However, establishing long-term therapy regimens with adequate dosages to minimize the negative consequences of over-activating autophagic pathways will represent a challenge. It is noteworthy that autophagy and other lysosome-targeting therapies can promote tumor development by boosting tumor growth, invasion, and resistance to treatment. Lysosomes deliver amino acids to tumor cells by degrading recycled intracellular materials and ingested extracellular proteins by autophagy and macropinocytosis, respectively. Moreover, cathepsins, a peptidase family in the lysosome, can degrade the extracellular matrix, stimulating epithelial-mesenchymal transition, a mechanism that has been widely known to promote invasion. In addition, drugs can be sequestered in lysosomes by active transport or passive diffusion, thereby lysosomes can contribute to chemoresistance [133].

Furthermore, it should be noted that most currently available therapeutics also target other biological processes beyond autophagy, emphasizing the need for innovative pharmacological agents with superior specificity, pharmacokinetics, and safety properties. Crucially, sensitive biomarkers will be required to assess the in vivo effectiveness of autophagy modulators.

### 4.3. Promoting Neuroprotection

#### 4.3.1. L-type Voltage-dependent Ca^2+^ Channel (L-VDCC)

The most well-studied polymorphisms are in genes that cause mitochondrial malfunction, which could increase the stress sensitivity of DA neurons due to their steadily increased energy needs and intracellular Ca^2+^ levels [134]. L-VDCC antagonists rescue SN neurons from MPTP-induced degeneration by reducing mitochondrial stress [135]. SN neurons become dependent on L-VDCCs and Ca^2+^ as they age, rendering them sensitive to neurotoxicity that can be avoided by L-VDCC antagonists. Moreover, α-Syn leads to Ca^2+^ dysregulation, and accordingly, α-Syn aggregation [136]. Studies found that patients treated with L-VDCC antagonists had a lower risk of PD. Furthermore, intracellular Ca^2+^ modulates microglia from the “rest” state to the “active” immune-effector state [137]. As a result, numerous medications targeting microglia L-type Ca^2+^ channels exhibited neuroprotective and anti-inflammatory proprieties through inhibiting microgliosis, which is a defining pathogenic feature of PD [138]. In PD, changes in the phenotype of microglia occur early and may be an initial mechanism that connects cognitive and motor impairments, protein aggregation, and neurodegeneration [139]. In mesencephalic neuron or neuron-glia mixed cultures, nimodipine, an L-VDCC antagonist, protected neurons against inflammation and MPTP toxicity in a microglia-dependent manner [140]. Nimodipine reduced microglia reactivity in the SNpc in a rat model of chronic inflammation with selective SN fragility, but had no neuroprotective benefit for DA neurons [141]. In contrast to several studies [135,140] that considered L-VDCC antagonists as broadly neuroprotective and anti-inflammatory agents, the first study to use genetic strategies to decrease microglial L-VDCC expression showed a noticeable increase in neuroinflammation and neurotoxicity following knockdown of caveolin-1 calcium channels (Cav1.2) on microglia [142]. Moreover, a phase III clinical study (NCT02168842) investigating isradipine, an L-VDCC blocker, showed that long-term therapy with immediate-release isradipine did not affect the clinical progression of early-stage PD [143].

#### 4.3.2. Glucagon-like Peptide-1 (GLP-1) Agonists

GLP-1 is an endogenous peptide hormone secreted by intestinal L-cells [144]. Under hyperglycemic conditions, it enhances insulin secretion from the pancreatic β-cells while decreasing glucagon secretion from α-cells, improving glycemic homeostasis and restoring sensitivity to insulin. Type 2 diabetes mellitus (T2DM) is a risk factor for neurodegenerative disorders. T2DM, AD, and PD share several pathophysiological features, such as oxidative stress, abnormal protein processing, inflammation, and insulin resistance, implying that effective T2DM drugs that positively modulate the gut–brain axis may be a treatment option for neurodegenerative diseases [145]. The GLP-1 receptor (GLP-1R) is found not just in the pancreas but throughout the body, including most areas of the brain. GLP-1R is a seven-transmembrane spanning class B G protein-coupled receptor. When activated on neuronal cells, it raises intracellular cAMP levels, resulting in protein kinase A (PKA) activation. It also causes the activation of the PI3K/AKT signaling pathway. These pathways modulate several downstream targets, including glycogen synthase kinase-3 beta (GSK3-β) and forkhead box protein O1 (FOXO1), which play essential roles in the pathological processes of PD, promoting an antiapoptotic cell survival pathway [146]. Furthermore, during a neuroinflammatory condition, PI3K/AKT signaling regulates NF-κB, in response to GLP-1R activation. NF-κB controls the activation of microglial cells and the release of pro-inflammatory cytokines TNF-α and IL-1β [147,148]. This reduces neuroinflammation, and because neuroinflammation is a hallmark in the etiology of PD, such GLP-1R-mediated activity may be advantageous.

Exenatide protected SH-SY5Y cells against hydrogen peroxide (H_2_O_2_)-induced cell death in a dose-dependent manner via GLP-1R activation [149]. Similarly, the incretin mimic geniposide was found to reduce H_2_O_2_-induced cell death in PC12 cells [150]. GLP-1R agonists showed cytoprotective and neuroprotective benefits in various in vitro and in vivo models. Exenatide completely reversed MPTP toxicity, raised the number of DA neurons and TH levels, and regulated inflammation [151]. Exenatide induced ciliary neurotrophic factor (CNTF)-mediated cell proliferation in mice, as demonstrated by GLP-1 overexpression in the hypothalamus and a loss of neuroprotective activity in GLP-1R knockout mice [145]. GLP-1R agonists dramatically enhanced the generation of doublecortin-positive immature neurons or progenitor cells in the dentate gyrus [152], which is an indirect marker of neurogenesis in the hippocampus. Neurite outgrowth is another benefit of the incretin signaling pathway. GLP-1 agonists have been demonstrated to increase nuclear respiratory factor 2 (Nrf2) expression. GLP-1R activation offered cytoprotective effects in PC12 cells and stimulated neurite outgrowth in a way comparable to nerve growth factor (NGF) [153]. These findings show that GLP-1R stimulation in neurotoxin-derived PD models improves DA cell survival and resolves the aberrant behavior. Moreover, this supports the concept that GLP-1R activation may have therapeutic potential for PD. As proof of this principle, 45 individuals with moderate PD and already receiving standard PD medication were randomly allocated to take subcutaneous exenatide injections in an open-label clinical study. The results revealed statistically significant and clinically major improvements in motor and cognitive symptoms. Exenatide was well-tolerated, with weight loss being the main adverse effect, which did not affect the trial outcome [154]. Regardless of the limitations of this study, advantages from exenatide treatment in both cognitive and motor skills continued in the follow-up study after a 12-month “wash-out period.” The results were encouraging enough that the same investigators initiated a double-blind clinical study (NCT01971242) assessing a sustained-release formulation of exenatide in a similar group of PD subjects over a similar duration. Findings mainly confirmed the conclusions from the prior open-label study. The investigators are now enrolling exenatide in a phase III study to evaluate efficacy over an extended period, to confirm its safety and tolerability in a wider patient group (NCT04232969). Another placebo-controlled trial evaluating subcutaneous injections of exenatide 2 mg once weekly for 48 weeks besides patient regular medication found that exenatide had positive effects on practically defined off-medication motor scores in PD that lasted beyond the period of exposure. However, it is unclear if exenatide impacts the underlying illness mechanism or just causes long-lasting clinical symptoms [155].

Two randomized, double-blind, placebo-controlled phase II clinical studies (NCT04269642 and NCT04154072) with another formula of exenatide were initiated. NCT04269642 was conducted to assess the efficacy and safety of sustained-release exenatide (PT320) in patients with early PD. PT320 demonstrated greater BBB penetration and improved patient compliance. NCT04154072 is an ongoing trial to evaluate the safety, tolerability, and efficacy of NLY01, a PEGylated version of exenatide. It has been demonstrated that PEGylation of substances increases their permeability across the BBB. Liraglutide, a new GLP-1 mimetic with a longer biological half-life compared to exenatide, has been accepted by the FDA to treat patients with T2DM and obesity; however, it is currently undergoing a phase II trial to assess its efficacy and safety profiles as a potential drug for PD treatment (NCT02953665). Oxyntomodulin, a natural GLP-1R/glucagon receptor (GCGR) dual agonist peptide, has previously exhibited greater efficacy in T2DM than a GLP-1 agonist alone and could be a potential candidate for this family of compounds directed to the gut-brain axis [156].

#### 4.3.3. Peroxisome Proliferator-Activated Receptors (PPARs) Agonists

PPARs are ligand-activated transcription factors of the nuclear hormone receptor superfamily that includes three subtypes: PPARα, PPARγ, and PPARβ/δ. PPARα/γ/δ stimulation lowers the triglyceride level while increasing insulin sensitivity and fatty acid metabolism, highlighting the regulatory role of the PPAR family in energy homeostasis and metabolic activity [157]. Many in vitro and in vivo studies reported promising neuroprotective properties of PPAR agonists. PPAR interacts with various domains of PGC-1α and PGC-1β (transcriptional co-activators) and regulates the expression of nuclear genes encoding enzymes participating in glucose metabolism. PGC-1α also interacts with PPARα, PPARδ, and other transcription factors to control mitochondrial biogenesis and respiratory functions in various tissues [158]. Pioglitazone (a PPARγ agonist) was found to protect DA neurons from MPTP by reducing MAO-B activity, in turn, preventing MPTP conversion to 1-methyl-4-phenylpyridinium (MPP^+^) [159]. It also slows microglia activation and decreases the inflammatory mediator levels, nitrotyrosine production, and the number of activated astrocytes in the brain [160]. Pioglitazone reduces oxidative damage and restores mitochondrial potential and DA neuronal function in the brain [161]. Nonetheless, evidence from a phase II clinical trial of pioglitazone (15 or 45 mg/day) implies that pioglitazone at these doses is unlikely to affect early PD development (NCT01280123) [162].

The PPARδ isoform is prevalent in the hypothalamus and is essential for brain homeostasis. PPARδ has also been identified as a powerful modulator of anti-inflammatory responses. L-165041, a PPARδ agonist, protected murine microglial cells against damage caused by radiation or inflammation. PPARδ suppresses NF-κB by binding with the p65 subunit and inhibiting the PKC/MEK1/2/ERK1/2/AP-1 pathways [163]. GW-501516, a highly selective PPARβ/δ agonist, and L-165041 rescue SH-SY5Y cells against MPP^+^-induced apoptosis through decreasing caspase-3 activation [164]. A recent study found that intra-cerebroventricular injection of GW501516 had protective activities in an MPTP-induced mouse model of PD [165]. GW501516 not only improved mobility impairment but also lowered DA neurodegeneration and inhibited activation of the nucleotide-binding domain and the leucine-rich-repeat-protein 3 (NLRP3) inflammasome in astrocytes but not microglia.

Meanwhile, PPARα agonists, such as fenofibrate and bezafibrate, have been studied in the same mice models. Fenofibrate rescued DA neurons in SN. Bezafibrate, nevertheless, did not demonstrate any markable neuroprotective effect, which was attributable to the fact that bezafibrate’s dosage was ten times lower than fenofibrate’s [166]. Non-steroidal anti-inflammatory drugs (NSAIDs), such as indomethacin, naproxen, and fenoprofen, have been shown to activate PPARα and PPARγ [167,168]. As a result, indomethacin and ibuprofen may exhibit neuroprotective effects against neurodegenerative diseases, such as PD. In mesencephalic cells, acetaminophen at 1 mM or ibuprofen at 0.1 mM concentrations was found to reduce the MPP^+^-induced DA neurotoxicity [169]. Similarly, in a mouse model of PD, indomethacin reduced microglial activation and lymphocyte infiltration, rescuing the DA neurons from MPTP-induced neurotoxicity [170]. Many other NSAIDs do not activate PPAR but promote neuroprotection in PD models via PPAR-independent pathways by reducing oxidative damage and NF-κB translocation. It has been reported that NSAIDs use lowers the risk of developing PD [171].

#### 4.3.4. PGC-1α

PGC-1 is the primary regulator of mitochondrial biogenesis. High levels of PGC-1α and PGC-1β are found in tissues with a high energy requirement and a high number of mitochondria, such as skeletal muscles and cardiac muscles [172]. Changes in mitochondrial biogenesis and dynamics were found to be directly linked to PD onset. This led to a thorough examination of the role of PGC-1s in the onset and progression of PD [173]. In vivo studies on transgenic mouse models of PD in which PGC-1α was either knocked out or genetically disrupted revealed an enhanced vulnerability to the neurodegenerative effects of MPTP and kainic acid, due to a lack of PGC-1α-dependent induction of the antioxidant response [174]. This led to DA neuron loss, a marked decline in mitochondrial biogenesis protein markers, and respiratory chain deficiency, resulting in PD onset [175]. A large cohort comparing PD patients and age-matched controls found two PGC-1α variants (rs6821591 CC and rs2970848 GG) related to PD onset [176]. Furthermore, the levels of PGC-1α and mitochondrial markers in PD patients were lower than in control patients and were adversely linked with disease severity. PGC-1α overexpression protected against neurodegeneration produced by classic toxin-induced models of PD in numerous in vitro and in vivo studies [177,178]. Treatment with phytochemicals, such as ferulic acid and resveratrol, restored mitochondrial dynamics via increasing PGC-1α expression in a mouse model of PD. This led to an increase in mitochondrial biogenesis and a decrease in ROS accumulation, which modulated the implicated mitochondrial dysfunctions [179,180]. PGC-1α also increases the production of antioxidant enzymes, including catalase, glutathione peroxidase, and manganese superoxide dismutase, which reduces oxidative damage [174]. Another study found that lower levels of PGC-1α in PD brain, neuronal cells, and PGC-1α knockout mice elevated α-Syn oligomerization and toxicity [181]. Thus, PGC-1α could be a key transcriptional coactivator implicated in neuroprotection in a variety of neurodegenerative disorders. Ambroxol, which is currently in phase II clinical trials as a disease-modifying agent in the treatment of PD (NCT02941822), modulated the mitochondrial content in primary cortical neurons by increasing the production of PGC-1α [182]. Thus, the restoration of PGC-1α represents a potential strategy for developing effective drugs to treat PD.

Parkin-interacting substrate protein (PARIS) accumulates in the human PD brain and regulates PGC-1α production. PARIS functions as a physiological transcriptional repressor of PGC-1α, suppressing the coactivator and its target genes [92]. The *PINK1*/*PRKN* axis mediates PARIS degradation by ubiquitination, and accordingly, regulates the levels of PARIS, ultimately affecting the levels of PGC-1α. Mutations in either *PINK1* or *PRKN* can disrupt this regulatory system, allowing PARIS to accumulate within neurons. PARIS overexpression impairs mitochondrial biogenesis, resulting in progressive DA neuron degeneration [30]. Thereby, *PRKN* is critical to PGC-1α to boost mitochondrial biogenesis via inhibiting PARIS. Aside from *PINK1* and *PRKN*, an interference with additional genes frequently altered in PD was demonstrated. One of these is *SCNA*, the α-Syn gene, because α-Syn upregulation and oligomerization negatively affect PGC-1α levels in the PD brain and cell culture models [181]. α-Syn affects mitochondrial function by decreasing the expression of the coactivator and associated target genes. The relationship between PGC-1α and α-Syn can affect PD progression. Therefore, an improved understanding of how the various players can work together to protect against neurodegeneration is needed.

#### 4.3.5. Iron Chelators

Iron accumulation in SN neurons of PD patients has been detected and assumed to be a disease-causing mechanism that has been connected to disease severity [183]. Overexpression of α-Syn increases the intracellular iron levels and iron redistribution from the cytoplasm to α-Syn-rich inclusions, leading to the acceleration of the α-Syn aggregation rate and fibril formation, most likely by increasing α-Syn protein translation [184]. Furthermore, iron can produce ROS [185]. As a result, removing iron from the SN may halt disease progression. Preclinical research suggests that BBB-permeable iron chelators could be used as disease-modifying agents in PD to remove excess iron [186]. Notably, serum iron levels have been established as a prospective marker for PD in a large Mendelian randomization study [187]. Treatment with deferoxamine (DFO) was reported to effectively ameliorate behavioral impairments and boost DA neuron survival in an MPTP-induced mouse model of PD [188]. Nevertheless, developing another synthetic metal chelator was necessary due to limited oral bioavailability of DFO, short half-life, and poor BBB penetration. Several 8-hydroxyquinoline analogs have also shown considerable promise in the treatment of neurodegeneration in PD. Clioquinol, a lipophilic iron chelator, reduced iron accumulation in the SN, inhibited iron-α-Syn interaction, and subsequently prevented α-Syn-related cell death, and the deterioration in motor and cognitive function in animal models of PD [189,190]. Another effective BBB-permeable iron chelator, VK-28, attenuated neuronal cell death caused by 6-OHDA in rats [191]. HLA-20 and M30 are neuroprotective agents with dual iron-chelating and MAO-B inhibitory action that combine the iron-chelating activity of the porotype VK-28 with the propargylamines moiety, which is known for its powerful MAO-B-inhibitory and neuroprotective effects. These compounds demonstrated iron-chelating efficacy equivalent to DFO, along with selective MAO-A- and MAO-B- inhibiting and protective properties [192,193]. Deferiprone (DFP) is another iron chelator with neuroprotective benefits in the MPTP-induced animal model of PD. In addition, unlike DFO, DFP can cross the BBB [194]. These findings inspired the launch of a double-blind, randomized, placebo-controlled pilot clinical study of DFP in PD to assess the drug safety profile, brain iron content changes, and PD clinical status. Findings demonstrated that short-term DFP therapy for PD patients was safe and could reduce iron in certain brain regions [195]. FAIRPARKII (NCT02655315) is a current randomized phase II study of conservative iron chelation therapy on a large population of PD patients. Many unresolved concerns with the iron chelation strategy remain, including the drug dose, lack of target engagement biomarkers, and the stage of disease chosen for patient enrollment. Whether removing iron will have any clinical or disease-modifying benefits is also unknown.

In conclusion, current attempts to develop neuroprotective therapies are based on the proposed roles of oxidative stress, metabolic dysfunction, and excitotoxicity in the degenerative process. Several agents designed for neuroprotective treatment of PD have shown great promise in the laboratory but failed to demonstrate any clinical benefits in recent high-profile clinical trials. This “failure to translate” is most likely related primarily to the currently incomplete understanding of the pathogenic mechanisms underlying PD and the over-reliance on data from toxin-based animal models when choosing the drugs that should be tested in humans. Repurposing existing drugs for use in PD (e.g., exenatide, ambroxol, and DFO) can be a promising technique for PD therapy with the benefit of reducing the drug development timeline, but it also requires more investigation.

### 4.4. Targeting Mutated Genes

#### 4.4.1. LRKK2 Inhibitors

Mutations of the *LRRK2* gene are reported in around half of all genetic variants discovered in databases of familial and sporadic PD cases. Furthermore, LRRK2-associated PD matches the symptoms, disease development, and age of onset of typical sporadic PD [196]. The increased activity of LRRK2 kinase was found to mediate PD-associated pathogenic phenotypes [196]. LRRK2 phosphorylates a subset of RAB GTPases that control inflammation, autophagy, lysosomal dysfunction, and neuronal differentiation [197]. LRRK2 inhibition attenuated shortening in neurite length in primary neuronal cultures from transgenic mice overexpressing *LRRK2* mutation G2019S or R1441G [198] and increased DA release and synaptic vesicle mobilization/recycling in R1441G mice. LRRK2 inhibition modulated the pro-inflammatory microglial signaling in rats [199]. The induced pluripotent stem cells (iPSC)-derived neural stem cells from PD patients harboring the LRRK2 G2019S mutation showed altered Ca^2+^ dynamics and mitochondrial DNA damage; however, all disease phenotypes were rescued via LRRK2 inhibition [200,201].

To date, there are four generations of LRRK2 inhibitors with different potencies, selectivity, oral availabilities, and brain penetration profiles. MLi-2 and PF-06685360 (also known as PFE-360) are potent and selective third-generation inhibitors with proper pharmacological properties [202]. It should be noted that LRRK2 is not a neurologically-specific protein. It is also abundantly found in kidney, lung, and immune cells, necessitating a thorough understanding of any potential safety risks of such drugs for translating LRRK2 inhibitors to the clinic [197]. Several research studies have implicated LRRK2 in infection, particularly in response to bacterial pathogens. LRRK2 contributes to the in vitro restriction of the enteric pathogen Salmonella by macrophages [203]. This conclusion was verified in vivo by showing that mice missing LRRK2 are more sensitive to peritoneal inflammation, resulting in poor Salmonella control and higher mortality in infected animals [204]. This study also evaluated the effect of the LRRK2 kinase inhibitor GSK2578215A in vivo, and observed that inhibiting LRRK2 kinase enhances mice susceptibility to Salmonella infection. Similarly, LRRK2 knockout mice were more susceptible to oral infection with the foodborne pathogen *Listeria monocytogenes* but not systemic infection [205].

Preclinical investigations revealed that some LRRK2 inhibitors cause morphological abnormalities and aberrant cytoplasmic buildup of lysosome-related lamellar structures in type II pneumocystis [206,207]. Nonetheless, relatively lower dosages of PF-06685360 and MLi-2, which significantly reduced LRRK2 in the brain, did not stimulate lung disease in non-human primates. Furthermore, the pulmonary disease caused by high-dose treatment was reversible after drug termination, with no deficits in lung function. Renal cells also express the highest level of LRRK2; therefore, it is no surprise that LRRK2 deletion has the most dramatic effect on the kidney [206]. In LRRK2−/− mice, loss of LRRK2 causes striking age-dependent accumulation and aggregation of α-Syn and ubiquitinated proteins in the kidney, although no apparent neuropathological changes were found in the brain [208]. Another issue with LRRK2 inhibitors is their impact on immunological homeostasis. The *LRRK2* mutation G2019S was linked to increased peripheral inflammation in asymptomatic carriers [209], indicating the possible anti-inflammatory effects of LRRK2 inhibitors. In phase I clinical trials, DNL201, an LRRK2 inhibitor, was safe and well-tolerated, with no significant side effects [210]. DNL201 phase Ib research (NCT03710707) is underway for mild-to-moderate PD patients with and without an *LRRK2* mutation [211].

#### 4.4.2. GCase Agonists

The most common genetic risk factor for PD is mutations in the *GBA* gene, encoding the lysosomal enzyme GCase. The mechanisms by which *GBA* leads to PD are not entirely understood. One of the possibilities is that GCase and α-Syn form a bidirectional self-propagating feedback loop. Loss of GCase activity results in α-Syn accumulation, leading to neurotoxicity via aggregate-dependent mechanisms. Furthermore, high levels of α-Syn delay both proteolytic and GCase function [212]. GCase transport from the endoplasmic reticulum to the lysosome is hindered by α-Syn until the neurodegenerative threshold is achieved [213,214]. Gene therapy, with adeno-associated viral vectors to transfer modified DNA to human cells, can restore GCase activity. The introduction of GCase by adeno-associated virus reduced the aberrant buildup of the hazardous lipid glucosylsphingosine and lowered the amounts of ubiquitin, tau, and α-Syn aggregates [215]. The benefit of gene therapy was that we could deliver a gene as an agent to a specific brain area to change altered function and cure PD while avoiding off-target consequences [216]. Virus delivery vectors can be used to deliver one of the crucial targets of PD neuronal regeneration, such as neurotrophic factor (e.g., glial cell line-derived neurotrophic factor (GDNF) and neurturin), or for the synthesis of neurotransmitters (e.g., aromatic amino acid decarboxylase, and TH/aromatic amino acid decarboxylase/GTP cyclohydrolase for prolonging the duration of L-dopa), and the potential proteins that might be a target to modulate via gene therapy (e.g., G protein-coupled receptor kinases) [217].

Isofagomine (afegostat tartrate, AT2101) was one of the first GCase chaperones to undergo clinical trials for Gaucher disease. However, additional advances for this indication were halted because isofagomine did not result in clinical improvement. Ambroxol is another example of a promising GCase chaperone, clinically used as a mucolytic drug, and can help transport the misfolded GCase protein to the lysosome. Ambroxol promotes autophagy functioning and increases enzymatic response in patients with GBA mutations [218], besides affecting the expression of PGC-1α (as previously stated) [182]. An open-label non-controlled clinical trial of oral ambroxol therapy confirmed that ambroxol has the potential to target the GCase pathway in PD patients with and without GBA mutations. Ambroxol increased GCase activity in the brain and modulated α-syn levels [219]. The effects of the GCase enzyme activator LTI291 were studied in a 1-month phase Ib trial, and around 40 GBA-PD patients took part in the study. No adverse events were reported, and the findings showed that the drug had good dose-dependent brain penetration [220].

To sum up, the identification of genetic variants that cause or increase the risk of PD has given researchers a new arsenal of possible therapies to explore in clinical trials. We highlighted two PD genes (*GBA* and *LRRK2*) linked to the lysosomal pathway and offered insight into the disease process. As previously stated, lowering LRRK2 activity or increasing the lysosomal activity of GCase are currently viable therapeutic options for the treatment of PD.

### 4.5. Targeting Neuroinflammation

#### 4.5.1. Phosphodiesterase 10A (PDE10A)

PDE10A is a striatal enzyme that hydrolyzes cAMP and cGMP in the medium spiny neurons [221]. PDE10A regulates DA signaling and many other brain activities, including ion conductance and synaptic plasticity [222]. In mouse models of PD, variations in cAMP/cGMP levels have been associated with the development of LID [223]. Additionally, 6-OHDA-induced lesions of nigrostriatal projections showed decreased striatal PDE10A expression. PDE10A deficiency was associated with DAT deficiency in the striatum in early de novo and early L-DOPA-treated PD patients, implying a link between PDE10A and DA function [223]. On the contrary, the PDE10A inhibitor papaverine reduced DA neuronal cell death via improving the expression of neurotrophic factors, such as brain-derived neurotrophic factor (BDNF), GDNF, and B cell lymphoma 2, all of which are regulated by PKA signaling. Papaverine also inhibited microglial activation and inflammatory gene expression, indicating that it could be used to treat neuroinflammatory disorders such as PD [224]. Strikingly, molecular imaging by positron emission tomography showed reduced PDE10A expression in mild-to-advanced _L_-DOPA-treated PD patients. As a result, PDE10A is a promising candidate sign of disease burden in individuals with early PD, with a diagnostic value similar to the gold standard, DAT molecular imaging. However, it is still unknown whether PDE10A is involved in the disease’s early stages [225].

#### 4.5.2. TLRs

TLR2 and TLR4 are increasingly being linked to PD. TLR2 levels were enhanced in postmortem brain tissue of PD patients. Furthermore, activation of neuronal TLR2 with the TLR2 agonist, PAM3CSK4, increases the levels of endogenous α-Syn concomitant with increased levels of the autophagy/lysosomal pathway marker p62 [226]. Rapamycin-induced autophagy or TLR2 signaling pathway blockage suppressed the TLR2-mediated increase in α-Syn in neuronal cell cultures [227]. Immunization with anti-TLR2 antibodies resulted in reduced α-Syn accumulation, microgliosis, pro-inflammatory cytokine release, and memory deficit in α-Syn overexpressing mice [228]. TLR4 antagonists, on the other hand, could prevent glia from overproducing inflammatory mediators and cytotoxins. However, they may have negative CNS effects by limiting glial phagocytosis, lowering protein clearance, and influencing myelination. Thus, selective TLR-4 agonists may be advantageous to increase the microglial phagocytic function and the clearance of aberrant α-Syn aggregates, but they can also trigger the release of inflammatory markers and cytotoxins. Accordingly, agonists that disingenuously induce the TLR 4 downstream myeloid differentiation primary response protein 88 (MyD88)-independent pathway should be improved and studied further, as such agents can enhance glial phagocytic activity without elevating cytokines and cytotoxins [229].

TLRs have been identified as critical pattern recognition receptors implicated in the identification of pathogen-associated molecular patterns and host-generated damage/danger-associated molecular patterns. As such, they can trigger the pro-inflammatory immune response by activating NF-κB and other transcription factors, resulting in neuroinflammation, which is found in both sterile and infectious CNS inflammatory disorders and may lead to neurodegeneration [230]. TLRs are expressed by a variety of immune cells in the CNS; however, excessive activation of these local immune cells results in a severe invasion of peripheral immune cells into the brain, which is harmful to the host and leads to a variety of neuroinflammatory illnesses [231]. Aggregated α-Syn oligomers caused microglial activation in vitro in a primary neuron-glia co-culture system, resulting in increased DA neurotoxicity. The mechanism by which aggregated α-Syn activates microglia remains unknown, but one rational notion includes α-Syn acting as a danger-associated molecular pattern and activating TLRs [232].

In brief, preclinical and clinical studies have provided evidence supporting the role of inflammation in the progression of PD. Furthermore, the inflammatory responses in PD have been linked to glial activation as well as peripheral immune cell infiltration; however, the association between these two different inflammatory pathways is still uncertain. These issues considerably hinder the improvement of PD-modifying therapeutics targeting inflammatory pathways. Moreover, the different states of microglia (activated or phagocytic) further contribute to the difficulty and complexity of manipulation of microglial responses in PD. A deeper knowledge of how these two activated phenotypes of microglia contribute to PD progression may pave the way for future therapeutic development.

### 4.6. Others

#### 4.6.1. Adenosine A2A Receptor (A2AR) Antagonists

A2AR is a non-DA target as it is specifically localized to the basal ganglia, whereas the indirect output pathway that influences the striato-thalamocortical loops crucial to the manifestation of the motor symptoms of PD [233]. High levels are located in the striatum, where they interact with dopamine D2 receptors [234]. Both have antagonistic effects on adenylate cyclase and cAMP synthesis. Activating A2AR suppresses dopamine D2 receptor signaling. Conversely, A2A antagonists boost the D2-dependent signaling cascade by inducing the expression of the immediate-early gene *c-fos* in the striatopallidal pathway [235]. Additionally, inhibition of A2AR reduces α-Syn-mediated neurotoxicity via modulating microglial reactivity and neuroinflammatory processes or by interacting with α-Syn, inducing conformational changes in the α-Syn aggregates [236]. Inhibition of A2AR modulates mitochondrial dysfunction by decreasing the excitotoxicity via interacting with NMDAR [236]. In dopamine-depleted rodent models of PD, A2AR antagonists, such as istradefylline, preladenant, KF17837, and Lu AA47070, reversed the motor impairments. In addition to ST1535 and PBF509, A2A antagonists modulated L-DOPA-induced rotational behavior caused by dopamine agonist or peripheral L-DOPA decarboxylase inhibitor (DCI) treatment in 6-OHDA-lesioned rats [233,237]. When istradefylline was administered alone in MPTP-treated non-human primates, it alleviated motor impairment. Furthermore, combination therapy with L-DOPA had a synergistic impact on the Parkinsonian scoring, and increased motor function was noticed compared to the benefits shown with L-DOPA/DCI alone, while substantially increasing the “ON” time in these animals [238]. In 6-OHDA-lesioned brains, prolonged treatment with four A2AR antagonists, caffeine, SCH 412348, istradefylline, or vipadenant, did not change the abnormal involuntary movement scale (AIMs). Meanwhile, chronic co-administration of SCH 412348 and L-DOPA/DCI did not aggravate or prevent the induction of AIMs typically generated by L-DOPA/DCI alone. These findings imply that, compared to L-DOPA, A2AR antagonists are less likely to cause dyskinesia, but they do not prevent the onset of expression of dyskinesia caused by L-DOPA [239].

Over 25 clinical trials have been performed to assess the safety profile and clinical effectivity of A2AR antagonists in PD patients, including eight phase IIb and III double-blind, placebo-controlled studies of istradefylline (KW-6002, > 4000 PD patients), one phase IIb trial assessing preladenant (SCH 420814, 253 PD patients), and one phase IIb trial evaluating tozadenant in 337 PD patients. All demonstrated motor improvements as an add-on therapy to L-DOPA in advanced PD patients [240,241,242,243]. After reviewing data from five paramount clinical trials (two phase IIb and three phase III trials, all of which used the change in the subjects’ percentage of daily awake time spent in the “OFF” state as the primary endpoint), the US FDA approved Nourianz^®^ (istradefylline) as add-on therapy to L-DOPA in adult patients with PD suffering from “OFF” episodes.

#### 4.6.2. 5-HT1A Receptor Agonists

Activation of 5-HT1A receptors can relieve LID in PD. However, staying within the safe therapeutic window in clinical studies has been problematic so far. Despite the promising outcomes in experimental models of PD [244], the nonselective 5-HT1A full agonist, sarizotan, had minor effects on dyskinesia compared to the placebo in phase III studies [245]. Furthermore, an increased dose in a phase II study deteriorated the motor activity [246]. Similar results were obtained with the 5-HT1A partial agonist, buspirone [247]. Two clinical trials are currently investigating its potential in LID along with its influence on motor function (NCT02617017 and NCT02589340). These trials aimed to (1) confirm the serotoninergic theory in hyperkinetic LID in PD patients, (2) conduct a phase III trial to test buspirone’s motor efficacy in improving LID vs. placebo, (3) examine a possible dose–effect relationship, and (4) test the hypothesis of a more effective therapeutic ratio using the association of buspirone and amantadine (NCT02589340) instead of a single drug; however, the latter study was terminated because of low enrollment. Eltoprazine, a 5-HT1A/B receptor agonist, relieved LID in a recent phase I/IIa trial without interfering with L-DOPA antiparkinsonian activity [248]. However, it deteriorated the antiparkinsonian efficacy of L-DOPA in experimental PD [249].

In conclusion, the chance of developing LID increases with the younger age of onset of PD, the severity of disease, and high L-DOPA dosage. Although the underlying mechanisms of LID are unknown, multiple therapeutics of various types have been attempted to treat it, including drugs acting on A2AR (e.g., istradefylline) or serotonergic receptors. It has been proposed that serotonergic transmission inhibits DA transmission. There have been instances of 5-HT agonists being used successfully to treat LID. However, these trials had a limited sample size and were generally uncontrolled.

### 4.7. Immunization

The immune system is activated in neuroinflammatory-based neurodegenerative disorders leading to increased neurotoxicity and degeneration. Hence, immune suppression could provide possible therapeutic advantages. Immunotherapy seeks to reduce inflammatory responses and create a neuroprotective state inside the CNS, avoiding additional neurodegeneration, which could be achieved by altering the patient’s immune system, followed by modifying the cerebral microenvironment. Stimulation of Tregs, a subset of T cells, inhibits the immune response and delays the cycle of neuroinflammation, leading to neuroprotection in PD patients. The GM-CSF is one example. GM-CSF is a cytokine with pro-inflammatory and anti-inflammatory modulating functions. It exhibits anti-inflammatory properties, owing to its induction of tolerogenic dendritic cells, leading to Treg activation and subsequent T cell-mediated tolerance [250]. GM-CSF-induced Tregs reduced microglial inflammation and preserved DA neurons in the MPTP-treated mice model [251]. In addition, GM-CSF treatment improved Treg functionality, implying that immunological modulation with GM-CSF enhanced Treg-mediated immune regulation, which is considerably reduced in PD patients, suggesting an effective therapeutic option. The immunological importance of GM-CSF has been investigated in animal models of PD and AD, and afterward in clinical trials in PD (NCT03790670) and AD (NCT01409915). In a phase I clinical trial, patients were given daily doses of sargramostim (GM-CSF) or placebo for eight weeks [252]. Sargramostim demonstrated a safe and well-tolerated profile, increasing Treg frequencies and function without affecting the levels of effector T cells (Teff cells). Furthermore, compared with pretreatment baselines and placebo-treated controls, sargramostim-treated patients had lower clinical ratings of disease severity, and magnetoencephalography revealed improved signaling in cortical regions relevant to motor function. An ongoing phase Ib trial in PD is currently evaluating the toxicity profile and potency of sargramostim doses and regimen length (NCT03790670). Due to GM-CSF’s short half-life, larger doses are required to maintain its plasma concentrations [253]. That may lead to mild-to-moderate side effects, including high levels of white blood cells, injection site reactions, and bone pain [252]. Studies on lipid nanoparticle-containing GM-CSF mRNA and a long-acting GM-CSF in animal models of PD were conducted to reduce these effects. These formulations enhanced Treg function, reduced microgliosis, and boosted DA neuron survival [254].

The neuropeptide vasoactive intestinal peptide (VIP) is a biological hormone that promotes neuroprotection by boosting Treg quantity and function, leading to inhibition of microglial stimulation, rendering it a promising immunomodulatory agent [255]. Due to its rapid metabolism and multiple recognition of VIP receptors 1 and 2 (VIPR1 and VIPR2), the development of a selective VIPR2 agonist with enhanced protease resistance and an extended duration of action is required to tackle these issues. LBT-3627, a VIPR2 agonist, reduced microglial activation and enhanced neuroprotection by producing an anti-inflammatory milieu, thus changing Th1/Th17-mediated pro-inflammatory immune responses in animal models [256,257]. Although selective VIP receptor agonists have yet to be evaluated in clinical trials of PD, further research is warranted because VIP is proven in animal models of PD to change systemic immunity to a less reactive and generally more anti-inflammatory phenotype.

The second strategy for immunotherapeutic intervention for PD patients is through passive and active immunotherapies, which are now in early-stage clinical trials to reveal which approach is more beneficial. Vaccination and active immunotherapy that promote host immunological responses provide prolonged biological clearance of target proteins by manipulating the host’s immune system without the need for repetitive administration. Active immunization techniques in PD must reprogram the host immune system’s self-tolerance to target antigens (α-Syn) and guarantee that enough antibodies are delivered across the BBB. Successful preclinical studies paved the path for a phase I trial of two anti-α-Syn vaccines, PD01A and PD03A. Both vaccinations did not exhibit major side effects at multiple doses [258].

DNA-based vaccination is an alternative technique that enhances the inhibition of total proteinopathy because it targets native protein substrates before aggregation. A nucleic acid vaccine (pVAX1-IL-4/SYN-B) boosted the antibody responses against α-Syn, provided DA neuroprotection, and improved behavioral functions in MPTP-lesioned mice [259]. However, using α-Syn-based animal models, no impacts on the α-Syn/target interaction were observed. When developing nucleic acid-based active immunization techniques, the lack of selectivity for pathologic α-Syn conformers and the potential of an excessive decrease of functioning monomeric α-Syn should be warranted.

Meanwhile, passive immunotherapies possess epitope selectivity of target antigens. However, repeated dosage administration could take several years. Most antibodies used in passive immunotherapies are stable, with long half-lives. Various passive immunotherapeutic agents are in different stages of clinical trials. PRX002/RG7935 (prasinezumab), the first passive immunotherapeutic agent, is now in a phase II trial (NCT03100149). PRX002 is a humanized IgG1 monoclonal antibody targeting C-terminal epitopes on α-Syn. Preclinical studies encouraged the launch of a phase I trial to investigate the pharmacological profile of PRX002 in healthy individuals [260] and also in idiopathic PD patients [261]. PRX002 was well-tolerated in both trials, with no major side effects or immunogenicity identified. Data from PD patients revealed that PRX002 had a sufficient CSF concentration to target the extracellular aggregated α-Syn in the brain [262]. BIIB054 is a fully human IgG1 monoclonal antibody that binds to an N-terminal on α-Syn. BIIB054 has a strong affinity for α-Syn aggregates (approximately 800-fold higher than the monomer) and effectively prevents α-Syn distribution in an α-Syn-based mouse model. BIIB054 was also beneficial in preventing motor deficits and sustaining striatal DAT levels [263]. Biogen Co. has released data from a phase I trial in healthy volunteers and PD patients. The side effects were minor and were thought to be unrelated to the therapeutic action. BIIB054 also demonstrated high amounts of bound serum α-Syn with a serum half-life of 28–35 days [264]. Biogen Co. subsequently launched a phase II clinical trial (SPARK) in January 2018 to investigate the safety, pharmacokinetics, and pharmacodynamics of BIIB054. However, the study did not reach its primary outcome for year one nor satisfied secondary outcome measures, so the development of BIIB054 (cinpanemab) for PD was halted, and the SPARK study was terminated. Other potential passive immunotherapeutic agents have undergone phase I clinical trials, such as Lu AF82422 and ABBV-0805 (previously BAN0805) (Table 2). A phase I trial is currently investigating the safety and tolerability of a single dosage of Lu AF82422 in healthy and PD patients in Japan (NCT03611569). ABBV-0805 preferentially targets oligomeric and protofibrillar α-Syn species in a transgenic model of synucleinopathy [265]. AbbVie Co. started a phase I study in 32 people with idiopathic, mild-to-moderate PD. Nevertheless, in July 2020, the study was withdrawn because of strategic considerations [266].

The several immunotherapeutic drugs now in clinical trials for PD have reawakened interest in the possibility of disease modulation. However, many of these attempts may be futile due to a basic lack of understanding of proteinopathy and its connection to disease progression and pathology. Furthermore, more focus should be directed toward finding reliable approaches to track and characterize disease development, particularly regarding α-Syn pathology, which is required to objectively determine the clinical potency of antibody-based therapy.

## 5. Stem Cell Research

Stem cells are unspecialized cells able to self-renew. Through mitosis, they can divide indefinitely to replenish other cell types in multicellular organisms. Stem cells are classified into (1) embryonic stem cells (ESCs) derived from embryos and (2) somatic or adult stem cells. The main variance between ESCs and somatic stem cells is that ESCs can convert into any cell type; meanwhile, somatic stem cells can only differentiate into similar cell types to the tissue of their origin. iPSC cells are genetically engineered stem cells created via boosting the expression of genes that cause cells to become pluripotent, eventually differentiating into any cell type in the body, including the DA neurons, and reverting to their original “stem cell” capabilities. Initially, this was accomplished by overexpressing four transcription factors (Oct4, Sox2, Klf4, and Myc), which may convert differentiated cells (e.g., fibroblasts, peripheral blood mononuclear cells) to become pluripotent (i.e., iPSCs) and exhibit stem cell traits similar to ESCs [267]. Although ESCs and iPSCs offer major benefits in terms of tissue availability over fetal midbrain tissues, both are very far from ethically neutral, especially ESCs, because the embryo is often murdered in the process of producing the ESCs [268].

Experimental research on iPSC-derived cells implanted into animal models of PD showed functional recovery and DA neuron survival [269]. Some groups adopted more “ethically” acceptable stem cell sources, such as parthenogenetic stem cells (derived from an unfertilized oocyte) [270]. However, this raises its own set of safety concerns due to the use of unfertilized eggs as a source of stem cells and the genetic abnormalities that this entails. Multiple laboratories have demonstrated effective implantation of dopamine neurons derived from human ESCs in rodent models, with the demonstration of both DA functional recovery and graft survival [271,272]. However, some of the potential issues with this approach are the risk of carcinogenesis and the development of teratomas, besides the ongoing proliferation of partially differentiated neural precursors included in the graft preparation [273]. Despite several neuronal populations in the brain that may produce dopamine, only the A9 group in the SNpc can innervate the striatum to recover function in PD. As a result, stem cell research has made concerted efforts to better understand the proper evolution of human-nigral DA neurons and discover the genetic markers that distinguish these particular neurons.

Furthermore, allogeneic or syngeneic transplantations currently confront two major challenges. One is the immune rejection of cells grafted into the brain, even if the cells are produced from the patient (i.e., autologous iPSCs); consequently, immunosuppression is required [274]. There is a hope that less-immuno-stimulatory cells will be accessible soon, for example, by selectively removing human leukocyte antigen (HLA) surface proteins, along with co-stimulatory molecules produced by the cell. Identification of the clustered regularly interspaced short palindromic repeats (CRISPR)/CRISPR-associated protein 9 (Cas9)-mediated genome editing technology (tool for precise modification of genome) has made this approach a more attainable goal [275,276]. The other major challenge is that grafted cells can become diseased through at least two distinct and non-exclusive pathways. Initially, because autologous iPSC-generated DA neurons are obtained from patients rather than healthy controls, the transplanted cells may be at risk of acquiring PD pathology, and heredity is thought to play a slight but not inconsequential influence on the total PD risk [277]. Additionally, even if allogeneic iPSC-derived DA neurons (e.g., genetically-related healthy donors) are eventually used, the normal neurons will be embedded in diseased brain tissue.

### Stem Cell-Derived Dopamine Replacement Therapies in PD Clinical Trials

Three pioneer clinical attempts have been performed and considered as critical guides to the field’s future steps (Table 3): (1) the European TRANSEURO research, which was developed to understand the possible advantages and disadvantages of fetal cell transplantation therapies [278]; (2) a single-center trial in Japan, where five patients were supplied with allogeneic iPSC-derived DA progenitors (from a stem cell bank with varying degrees of HLA matching to the hosts) to evaluate safety and quality of the technique (Clinical Trial: R000038278) [279]; and (3) in 2020, the first case report of a patient with PD who received midbrain DA progenitors derived from autologous iPSCs grown from the patient’s fibroblasts was published [280]. This latest study demonstrated that the used method was safe, the transplant did not create any masses, and that it was clear from any undifferentiated iPSCs. However, the DA neuron survival in the transplant appeared to be suboptimal, a result thought to be linked to how the DA neurons were generated, according to the published data. The differentiation protocol appeared inferior to that published by other groups. Finally, the International Stem Cell Corporation (ISCO) announced the successful completion of its dose-escalating phase I clinical trial (NCT02452723) evaluating the safety, tolerability, and preliminary efficacy of its lead candidate, ISCO human parthenogenetic neural stem cells (ISC-hpNSC) for the treatment of PD. In all cohorts, there have been no serious adverse effects related to the transplanted ISC-hpNSC cells [281].

Despite lacking the scientific rigor of real trials, individual case studies can be valuable; however, they must never be over-interpreted. For example, in the case report mentioned above, no declaration can be concluded about efficacy because there appeared to be a placebo effect due to the patient’s report of notable improvements on subjective quality of life scale, but without major changes in dopamine brain imaging or on more objective clinical scales. Finally, only properly planned double-blind placebo-controlled trials can ever demonstrate efficacy [282,283]. The safety of the transplanted cells, and the avoidance of unfavorable proliferation and tumors, will always be the top priorities. Suicide gene techniques (e.g., herpes simplex virus-1 thymidine kinase with ganciclovir) can destroy tumor-initiating cells following grafting; however, this alone is insufficient to protect against the danger of teratoma formation [284]. To exclude undifferentiated pluripotent stem cells, unique cell surface markers for stem cells, such as SSEA-3, SSEA-4, TRA-1-60, and TRA-1-81, have been used [285]. It has also been discovered that simply introducing an antimitotic at a specific point in the differentiation process is effective [286].

## 6. Growing Orientation: Biomarker Discovery

Although many breakthroughs in PD therapy have been accomplished, as summarized in Table 4, the discovery of biological markers that can offer more accurate diagnosis at an early stage of PD remains a significant unmet clinical need to halt PD progression. Given the lack of a definitive test for early PD diagnosis, molecular biomarkers are being researched as potentially useful clinical tools. Biochemical biomarkers can be studied in both CSF and blood; proteins and peptides reflecting specifically brain-derived activities can diffuse into the CSF in PD. In addition, many of the biochemical alterations are reflected in the CSF [287]. For example, the release of the primary astrocyte intermediate filament GFAP from brain tissue into the bloodstream is thought to occur when the BBB is disrupted or when astrocytic structural integrity is lost. When the astrocytes are damaged, GFAP breakdown products are released into the blood and continue to pursue the CSF flow [288]. GFAP levels in CSF are typically tested using an in-house enzyme-linked immunosorbent assay (ELISA) kit based on a specific antibody, with a low threshold of 70 ng/L and intra- and inter-assay coefficients of variation of 4% and 8%, respectively [289].

DJ-1a is another biological biomarker. The *PARK7* gene encodes this parkinsonism-associated deglycase. DJ-1 expression is specifically enhanced in oxidative stress, rendering it a biomarker for PD. However, because the DJ-1 level is elevated in erythrocytes in case of hemolysis or erythrocyte contamination, and can substantially affect the DJ-1 levels in CSF and plasma, assessment of erythrocyte contamination or hemolysis is required during DJ-1 measurements [292]. BDNF is another case. It is involved in neuronal survival regulation; decreased BDNF expression in the SN is related to DA degeneration. As a result, BDNF may be used as a biomarker in PD. In PD patients, the amount of BDNF in their CSF correlates with cognitive ability. The ELISA technique may be used to measure BDNF levels [293].

Neurofilaments are crucial intrinsic components involved in neuronal integrity and nerve impulse transmission along the axon. The presence of neurofilament light chain proteins in CSF is a symptomatic sign of neuronal degeneration, particularly the abnormally phosphorylated neurofilaments, which have been detected in PD linked with LBs [294]. An alternative biochemical indicative biomarker in PD is neuromelanin. Dying neurons in PD discharge neuromelanin, initiating a destructive cycle of neuroinflammation that usually leads to neuronal death. Thus, neuromelanin concentrations are also used to indicate SN damage status, and accordingly, could be considered as a biomarker for PD [295]. Lysosomal malfunction is widely being identified as a crucial phenomenon in the etiology of PD. The presence of lysosomal enzymes, such as GCase and cathepsin in the CSF, might be used as a biomarker for PD. However, CSF lysosomal enzyme activity alone cannot distinguish PD from other disorders. As a result, combining CSF lysosomal markers with CSF α-Syn aggregates or any signs of mitochondrial impairment, neuroinflammation, or other pathogenic proteins in PD may allow for a more definitive diagnosis [296].

In PD, miR-124 has a role in neuronal development, synapse architecture, neurotransmission, and regulating cell survival, autophagy, and mitochondrial dysfunction. According to a recent study, plasma levels of miR-124 in PD patients were considerably lower than in controls. As a result, miR-124 has the potential to be used as a diagnostic biomarker in PD [297]. Conversely, insulin-like growth factor 1 (IGF-1) serum levels were considerably higher in patients with undiagnosed PD at the time of assessment compared to healthy controls, indicating that serum IGF-1 can serve as a possible marker for idiopathic PD in the early stages of the disease. However, it should be noted that the PD cohort had a limited sample size and was carefully chosen in terms of concurrent treatments and comorbidities. As a result, while the findings may be only partially relevant to the general population, they demonstrate elevated blood IGF-1 levels in the very beginning stages of PD [298].

Inflammatory biomarkers are among the molecular biomarkers in PD. Fractalkine (CX3CL1) is an inducer that permits neurons and microglia to interact to control inflammation; low levels of fractalkine correlate with neuroinflammation. Thus, the fractalkine level is linked to the severity of illness and progression of PD [299]. Another inflammatory biomarker is neurosin. Neurosin is a serine protease, which is expressed predominantly in the brain and capable of hydrolyzing α-Syn [300]. Lowered neurosin levels have been observed in PD. The possible relationship between neurosin and α-Syn was examined in vivo using an in-house direct ELISA and commercial sandwich ELISA kit to assess CSF concentrations of neurosin and α-Syn in PD patients [301]. It is noteworthy that strict quality control measures and enough attention to covariance are crucial for lowering false-positive rates. Technical aberrations, such as batch effects (differences in the same sample detected at various times) and site effects (data collected from different machines may not be measured consistently), can readily confound ELISA and multiplex test findings. Ignoring variables for inflammation such as blood counts, BMI, sex, and chronic stress levels might result in erroneous conclusions. In addition, regardless of health state, inflammation levels are expected to fluctuate throughout time due to environmental and circadian rhythm variations. Measures taken at a single point in time may be deceptive. Longitudinal studies with several time-point assessments of inflammation in peripheral tissues are thus required to adequately quantify the inflammatory changes [302].

In addition to molecular biomarkers, a genetic demonstration can be used as a medical diagnostic sign of PD. Mutations in *α-Syn*, *PRKN*, *PINK1*, *DJ-1*, *LRRK2*, and *GBA* can be detected by analysis of global gene expression with DNA microarrays (Table 5) which has been performed in the peripheral blood of PD patients [303,304] and successfully serve as genetic biomarkers.

## 7. Conclusions

In conclusion, many significant breakthroughs have been made in our understanding of the etiology and pathogenesis as well as the pathophysiology of the basal ganglia in PD. These are paving the way for the development of innovative medicines that address some of the disorder’s most pressing unmet requirements. Some current medications may have the power to modify the disease, but our ability to test them correctly is restricted. A variety of non-dopaminergic medicines, including β-adrenergic, serotoninergic agonists, and adenosine A2a antagonists, are under late-stage development for PD and may help motor symptoms and consequences. Currently, the recent focus on non-motor symptoms in PD might speed the discovery of medications to address these critical features of the advanced PD. Furthermore, contemporary PD research trends have focused on customized treatments designed to restore the molecular, morphological, and functional integrity of disease-specific brain circuits. Significant technical breakthroughs in gene editing approaches, DBS devices, software, and neuroimaging, together with increasing knowledge of the methodological difficulties that have limited PD research in the past, have resulted in novel pharmacotherapeutic and non-pharmacological strategies that are currently being evaluated. In addition to that, advances in biomarker research and the identification of robust, presumably multimodal, markers of pathogenesis and disease progression are critical for the successful conduct of PD clinical trials aimed at filling a long-standing gap in disease-modifying, individually tailored treatment options.

## Figures and Tables

**Table 1:** Current approaches used in PD management.

Symptoms	Mode of Action	Therapeutics	References
Motor	Dopamimetic drugs	L-DOPA Dopamine agonists (ropinirole, pramipexole)	[17]
	Prevention of L-DOPA/dopamine breakdown	Decarboxylase inhibitors (carbidopa, benserazide); MAO-B inhibitors (selegiline, rasagiline, safinamide); COMT inhibitors (entacapone, opicapone)	[8]
	Glutamate inhibition	Amantadine	[18]
	Restoration of the balance between dopamine and acetylcholine	Anticholinergics (benztropine, procyclidine, trihexyphenidyl)	[13]
	Surgery	Deep brain stimulation; MRgFUS	[14,15]
Non-motor		Depending on the symptoms, antidepressants, cholinesterase inhibitors, or sedative agents may be used	[16]

PD: Parkinson’s disease, MAO-B: monoamine oxidase-B, COMT: catechol-O-methyl transferase, MRgFUS: magnetic resonance-guided focused ultrasound ablation.

**Table 2:** Clinical trials of immunotherapeutic interventions involved in PD management.

Therapeutic Class	Agent	Target/Mode of Action	Clinical Trial ID	Stage	Status
Immunomodulatory	GM-CSF	Enhances Treg quantity and functionality, with no impact on the levels of effector T (Teff) cells	NCT03790670	Phase Ib	Recruiting
	LBT36	Increases Treg abundance and performance, decreases microglial activation by altering Th1/Th17 cytokine responses	Not available	Not available	Not available
Active immunization	PD01A	C-terminal α-Syn mimicking peptide vaccination	NCT02618941	Phase I	Completed
	PD03A	C-terminal α-Syn mimicking peptide vaccination	NCT02267434	Phase I	Completed
	pVAX1-IL-4/ SYN-B	A nucleic acid vaccine that increases antibody titers against α-Syn	Not available	Not available	Not available
Passive immunotherapy	Prasinezumab	A humanized IgG1 monoclonal antibody targeted towards C-terminal epitopes on α-Syn	NCT04777331	Phase IIb	Recruiting
	BIIB054	A fully human monoclonal antibody directed against N-terminal α-Syn aggregate species	NCT03318523	Phase II	Terminated
	Lu AF82422	A humanized monoclonal IgG1 antibody targeting the C-terminal of α-Syn	NCT03611569	Phase I	Completed
	ABBV-0805	A humanized monoclonal antibody targeting α-Syn oligomeric and protofibrillar α-Syn species	NCT04127695	Phase I	Withdrawn

Treg: regulatory T cells, Th: T helper, IgG1: immunoglobulin G1, α-Syn: alpha-synuclein.

**Table 3:** State of stem cell-derived dopamine replacement therapies.

Sponsor	Therapeutic/Type of Cell	Stage	Clinical Trial (ID)
TRANSEURO	Human fetal ventral mesencephalic tissue	Phase I	NCT01898390
Kyoto University Hospital	Allogeneic iPSC-derived DA progenitors	Phase I/II	R000038278
National Institutes of Health	Autologous iPSC-derived dopamine progenitor cells	Case report	
ISCO	ISCO human parthenogenetic neural stem cells (ISC-hpNSC)	Phase I	NCT02452723

iPSC: induced pluripotent stem cell, DA: dopaminergic, ISCO: International Stem Cell Corporation.

**Table 4:** Different approaches for PD management.

Therapeutic Class	Approaches	Drugs Penetrating BBB	Ref
α-Syn aggregation inhibitors (NPT200-11, NPT088); Antisense oligonucleotides; β2AR agonists (salbutamol); LAG3 receptor	Targeting α-Syn	NPT200-11; NPT088; Anle138b; Salbutamol	[49,50] [53,57] [61] [69]
mTOR signaling pathway (rapamycin); Inhibitors of c-Abl (nilotinib and bafetinib); ROCK inhibitors (simvastatin and lovastatin); ASMase	Enhancing autophagy	Rapamycin; Nilotinib; Simvastatin; Lovastatin	[290] [101] [116,117] [129,130]
L-VDCC (isradipine); GLP-1 agonist (exenatide and oxyntomodulin); PPAR agonists (pioglitazone); PGC-1α activators (ambroxol); Iron chelators (DFO)	Promoting neuroprotection	Isradipine; Exenatide; Oxyntomodulin; Pioglitazone; Ambroxol; DFO	[143] [156,291] [162,166] [182] [195]
LRRK2 kinase inhibitors (MLi-2, PF-06685360, and DNL201); GCase agonists (isofagomine and ambroxol)	Targeting mutated genes	MLi-2; PF-06685360; DNL201; Isofagomine	[202,211] [220]
PDE10A inhibitors (papaverine); TLRs (anti-TLR2 antibodies)	Targeting neuroinflammation	Papaverine	[224] [228,229]
A2A receptor antagonists (istradefylline and tozadenant); 5-HT1A receptor agonist (buspirone and eltoprazine)	Others	Istradefylline; Tozadenant	[240,241,242,243] [248]
Immunomodulatory (sargramostim); Active immunization (PD01A and PD03A); Passive immunotherapy (prasinezumab and cinpanemab)	Immunization	Sargramostim; PD01A, PD03A; Prasinezumab; Cinpanemab	[253] [258] [260]
Human parthenogenetic neural stem cells (ISC-hpNSC); Human fetal ventral mesencephalic tissue (hfVM); Allogeneic/autologous iPSC-derived DA progenitors	Stem cell therapy	NSCs can be delivered trans-cranially	[281] [278] [279,280]

α-Syn: alpha synuclein, LAG3: lymphocyte-activation gene 3, mTOR: mammalian target of rapamycin, ASMase: acid sphingomyelinase lysosomal enzyme, L-VDCC: L-type voltage-dependent Ca^2+^ channel, GLP-1: glucagon-like peptide-1, PPAR: peroxisome proliferator-activated receptors, PGC-1α: peroxisome proliferator-activated receptor gamma coactivator-1alpha, GCase: glucocerebrosidase, PDE10A: phosphodiesterase 10A, TLRs: toll-like receptor, A2A: adenosine, BBB: blood-brain barrier, NSCs: neural stem cells.

**Table 5:** Proposed molecular biomarkers for PD diagnosis.

Type	Name	Role/Significance	Measurement	References
Biochemical biomarkers that can be investigated either in CSF or blood	Glial fibrillary acidic protein (GFAP)	This brain-specific protein and its breakdown products can serve as possible early biomarkers for PD	Detected via an in-house ELISA kit based on antibodies, with a sensitivity of concentration 70 ng/L and intra- and inter-assay coefficients of variation of 4% and 8%, respectively	[288,289]
	DJ-1	DJ-1 expression is implicated during oxidative stress	During measuring DJ-1 content in plasma and CSF, an assessment of contamination or hemolysis of erythrocyte is required	[292]
	Brain-derived neurotrophic factor (BDNF)	Reduced expression of BDNF within the SN serves as a potential biomarker for PD	Can be determined by ELISA	[293]
	Neurofilament light chain protein (NFL)	Recognized in PD associated with LBs	Measuring NFL increasing levels is a very sensitive method to assess aggressive neuronal death	[294]
	Neuromelanin	Released from dying neurons	Can be measured by MRI techniques	[295]
	CSF α-Syn with lysosomal enzymes	The combination of CSF lysosomal enzymes, such as cathepsin and GCase in the CSF with CSF α-Syn aggregates facilitate an improved accuracy of diagnosis	Detected by protein-misfolding cyclic amplification and real-time quaking-induced conversion	[296,305]
	miR-124	Plasma levels of miR-124 were markedly reduced in PD patients.	Detected in plasma	[297]
	Insulin-like growth factor 1 (IGF-1)	Potential marker for idiopathic PD in early disease stage	Detected in serum	[298]
Inflammatory	Fractalkine	Low levels of fractalkine associated with neuroinflammation	Detected in serum	[299]
	Neurosin	Can cleave and degrade α-Syn; decrease in neurosin levels have been reported in PD	Identified by Northern blotting/investigated in vivo using a commercial sandwich ELISA kit or a direct ELISA	[301]
Genetic	Mutations in *SCNA*, *PRKN*, *PINK1*, *DJ-1*, *LRRK2* and *GBA*	Triplication of the *SCNA* causes a two-fold increase in α-Syn expression.	Analysis of global gene expression with DNA microarrays has been performed in the peripheral blood of PD patients	[303,304]

CSF: cerebrospinal fluid, α-Syn: α-synuclein, ELISA: enzyme-linked immunosorbent assay, SN: substantia nigra, PD: Parkinson’s disease, miR-124: microRNA-124, *PRKN*: Parkin, *DJ-1*: Daisuke-Junko-1, *LRRK2*: leucine-rich repeat kinase 2, *GBA*: glucocerebrosidase, *PINK1*: PTEN-induced kinase 1.

## Data Availability

Not applicable.
